# A systematic review and meta-analysis of the epidemiology of Leptospirosis in HIV uninfected and in people living with HIV from the Southern African Development Community

**DOI:** 10.1371/journal.pntd.0010823

**Published:** 2022-12-12

**Authors:** Isac Rodrigues Comia, Regina Daniel Miambo, Emília Virgínia Noormahomed, Manuel Mahoche, Alberto Pondja, Robert Turner Schooley, Constance Benson, Jahit Sacarlal

**Affiliations:** 1 Faculty of Health Sciences, Lúrio University, Nampula, Mozambique; 2 Department of Microbiology, Faculty of Medicine, Eduardo Mondlane University, Maputo, Mozambique; 3 Department of Para-Clinics, Faculty of Veterinary, Eduardo Mondlane University, Maputo, Mozambique; 4 Mozambique Institute for Health Education and Research (MIHER), Maputo, Mozambique; 5 Department of Medicine, Division of Infectious Diseases and Global Public Health, University of California, San Diego, California, United States of America; Al-Jouf University College of Pharmacy, SAUDI ARABIA

## Abstract

**Background:**

Leptospirosis is an occupational, neglected febrile disease of bacterial origin transmitted between humans and animals. In this manuscript we summarize available data on *Leptospira* infection in HIV uninfected and in people living with HIV from the Southern African Development Community (SADC) countries, identifying gaps in knowledge and recommend future research priorities.

**Methodology:**

Articles published between 1990 and 2021 were accessed by an online search of Google Scholar and Medline/PubMed performed between February 2020 and July 2022. The STATA program was used for the Meta-analysis. Pooled prevalence values with 95% confidence intervals and heterogeneity were determined.

**Results:**

Thirty studies from eight SADC countries, reporting the prevalence on *Leptospira* were reviewed. A pooled prevalence of 19% (CI: 13–25%), a heterogeneity level of 96% and index score ranging from 2 to 9 was determined. Only four (4) studies reported HIV co-infection status. Three species of *Leptospira* (*Leptospira interrogans* (4), *L*. *kirschneri* (3), *Leptospira borgpetersenii* (1) and 23 serogroups were identified. The most frequently reported serogroups were Icterohaemorrhagiae (13), Grippotyphosa and Australis (10) followed by Sejroe (8).

**Conclusion:**

Studies on human leptospirosis in the SADC region are scarce, especially in people living with HIV. Additional studies aimed at determining the prevalence and the role of the pathogen in people living with HIV, including detailed clinical, molecular and demographic data are recommended.

## Author summary

The SADC region is severely affected by infections such as HIV, tuberculosis and malaria, which divert attention from several other diseases with major public health impacts. These critically neglected tropical diseases, include leptospirosis, cysticercosis, onchocerciasis, lymphatic filariasis, intestinal parasites and schistosomiasis among others. Leptospirosis is often misdiagnosed as one of several other febrile diseases including malaria, brucellosis and rickettsiosis because of similarities in symptoms. It is also likely underdiagnosed because of the complexity of laboratory diagnostic methodologies. Our review found that there is a scarcity of studies on leptospirosis, and lack of standardization tools for data collection, especially in the framework of leptospirosis in people living with HIV. In this review we summarize the pooled prevalence of leptospirosis in the SADC countries and the reported circulating serovars and species of *Leptospira* and uncover gaps in knowledge about the epidemiology of the pathogen. Misconceptions about the prevalence of leptospirosis among the scientific community, health care providers and policy makers coupled with complexity of clinical and laboratory diagnosis have resulted in a systematic underestimation of the public health impact of this important neglected pathogen.

## Introduction

Leptospirosis is a (re-)emerging, neglected, zoonotic bacterial disease caused by spirochetes belonging to the genus *Leptospira*. The disease is geographically widely geographically distributed and constitutes the leading zoonotic cause of morbidity and mortality worldwide, with approximately 1.03 million cases and 58,900 deaths per year [1,2]. The highest incidence of leptospirosis per 100,000 of population in the World were from Africa (95.5) followed by the Western Pacific (66.4) and the Americas (12.5) [3]. The disease thrives in countries with humid and tropical climates, poor sanitation, close contact with animals, heavy rains and floods combined with scarce health resources, all factors that favor the onset and spread of the bacteria [1,2,4–6]. Among livestock leptospirosis causes abortion, reproductive failure, premature birth or stillbirth, and reduces milk production each of which lead to monetary losses [1,7,8].

Several genospecies of *Leptospira* that infect humans are categorized as pathogenic. These include *Leptospira interrogans*, *Leptospira kirschneri*, *Leptospira borgpetersenii*, *Leptospira santarosai*, *Leptospira noguchii*, *Leptospira weilii*, *Leptospira alexanderi and Leptospira alstoni*. Saprophytic species that do not infect humans are also referred as "non-infectious" species [1,9,10]. Within the pathogenic species, 30 serogroups and more than 300 serovars were isolated based on serological phenotype analysis using, respectively, Microscopic Agglutination Test (MAT), Cross Agglutination Absorption Test (CAAT) [1,11,12] and the Polymerase chain reaction (PCR) analysis [1].

*Leptospira* species can be found in urine, kidney, genitals or other tissues of wild and domestic mammals. Rodents are often reported as the main reservoirs in urban areas [8,13], while dogs predominate in rural areas [4,14,15]. Livestock species such as cattle and pigs serve as carrier hosts in both rural and semirural areas [7,16–19]. Human infections are due to direct contact of injured skin or mucous membranes with contaminated urine, tissues or organs of infected animals. Contaminated soils and water can also serve as sources of infection [7,14,16]. Therefore, leptospirosis can affect a large number of at risk humans in a population [1,2,8].

In terms of clinical presentation, infected patients may be asymptomatic or have symptoms. Those with symptoms most frequently present with a febrile syndrome. These variations in clinical presentation may be attributed to individual immunological and genetic characteristics of the host, and to the pathogenicity and virulence of the bacteria, which are associated with specific surface proteins and toxin production [20–22]. The acute stage of the disease is accompanied by varying symptoms and signs such as fever, headache, myalgia, arthralgia, chills, nausea, abdominal pain, diarrhoea, cough, conjunctivitis and skin rashes which may appear 2 to 20 days after exposure [23]. Subacute and chronic complications as well as long-term sequelae may also occur [1]. In about 10% of infected patients with pathogenic serovars, the symptoms may progress to fulminant leptospirosis, known as Weil’s disease, characterized by multiorgan dysfunction with pulmonary haemorrhage, renal and liver impairment [2,24]. In tropical countries the nonspecific symptoms of fever, myalgia and arthralgia often lead to misdiagnosis with other endemic febrile diseases such as malaria, dengue, brucellosis, rickettsiosis, typhoid fever and babesiosis [25]. There is general agreement on leptospirosis treatment which includes administration of specific antibiotics, though in some cases the disease can resolve spontaneously without specific treatment [22].

There is a controversy regarding the clinical presentation and outcomes of leptospirosis in people living with HIV (PLHIV). Some authors argue that the clinical manifestations and severity of disease differ little from those in immunocompetent patients [26].

Globally, there were approximately 37.7 million people infected with HIV in 2020 [27]. The Southern African Development Community (SADC), comprising 16 countries has the highest morbidity rates of HIV/AIDS, with approximately 26 million people living with the disease in 2017. Most countries except Comoros, Seychelles, Madagascar and Mauritius register elevated morbidity and mortality rates [28].

It is well documented that HIV infection and immunodepression (expressed by CD4 cell count) may favor the acquisition and progression of tuberculosis. Much attention has been afforded to the diagnosis, treatment and control of other co-infections such as malaria that can be worsened by HIV infection [2,29,30]. Indeed, malaria which is the leading cause of mortality in SADC countries causing up to 47% of infectious deaths in this region, is often mistaken for leptospirosis [25,31,32].

Because of the possibility that leptospirosis may negatively impact SADC inhabitants, especially in the context of HIV, malaria, tuberculosis, and other neglected tropical diseases in the region, we conducted the present systematic review and meta-analysis. The aim of this study was to summarize and critically review available information on clinical and epidemiological features of leptospirosis, including diversity of *Leptospira* infection in HIV uninfected people and in PLHIV. From these data we hoped to uncover gaps in knowledge, develop recommendations for future studies with a view to clarify the clinical, epidemiological, and molecular aspects of this zoonotic disease.

## Methods

The information reviewed in this manuscript was reported following the Preferred Reporting Items for Systematic Reviews and Meta-analyses (PRISMA) guidelines [33]. We wished to sumarize available data on the prevalence of human leptospirosis, study population characteristics and identified genotypes or serovars of *Leptospira* in HIV uninfected patients and in PLHIV from SADC region. This region is composed of 16 countries including, Angola, Botswana, Democratic Republic of Congo, Lesotho, Madagascar, Malawi, Mauritius, Mozambique, Namibia, Seychelles, Comoros, Swaziland (Eswatini, since 2018), South Africa, Tanzania, Zambia and Zimbabwe [34].

### Search in electronic databases

The international electronic databases of PubMed (Medline), Scopus, Science Direct and Google Scholar (grey literature) were searched for relevant articles published between 1990`s through 2021 using medical subject headings (MeSH) and the following keywords combinations: "*Leptospira*" OR "Leptospirosis" AND "individual SADC countries". Retrieval of articles in all selected databases was done between February 2020 and July 2022. The selected articles were then entered into EndNote X8 software and duplicates were eliminated.

### *Leptospira* case definition

Negative case: participants with paired serum samples with the lack of a four-fold rise in the MAT titer with titers <1:800 in both samples or patients with a single serum sample and a reciprocal MAT titer ≤ 1:800.

Probable case: persons meeting the suspected case definition criteria with a positive ELISA IgM and any single reciprocal MAT titer ≥1:800.

Confirmed case: persons meeting the suspected case definition criteria with a positive real-time PCR assay for pathogenic *Leptospira* spp. in blood and/or a positive MAT as described above [35–38].

### Study inclusion and exclusion criteria

Articles written in English or Portuguese were selected for this study fulfilling one or more of the following criteria:

- Reporting on the prevalence of *Leptospira* in SADC countries;
- Reporting on *Leptospira* infections in PLHIV and on HIV uninfected patients;
- The use of a confirmative diagnostic test for *Leptospira;*
- Being either a cross-sectional, case-control, cohort, prospective, or a retrospective study.

All other studies were excluded, including review studies and case reports.

### Study selection and data extraction

Two authors (IRC and RDM) participated independently in the extraction and selection of the articles obeying the following stages: pre-selection of articles based on the information given in the title; full reading of the abstract and search of evidence within the search terms. Articles were discussed and a consensus was resolved by joint interpretation of contents by both authors. At the end of the literary search, author EVN reviewed all the articles including table presentations, interpretation and intervened in case of lack of consensus between authors.

### Quality of the studies

The quality of studies was evaluated based on the instructions of the standard quality assessment criteria for evaluating primary research papers [39]. These criteria include 10 items with a score of 0 for ‟Noˮ and 1 for ‟Yesˮ. Study quality score is expressed as a percentage calculated by summing up the score and dividing the sum by ten (10). The total score in all items generated an overall quality index that could range from 0 to 10. The median score was calculated based on the number of “Yes” scores obtained for each article and divided by the total number of the studies/articles. The median score obtained was 6.7. Studies were classified as high quality with a score above the median (6.7) and as low quality with a score below the median. The following questions were accessed for each selected study:

- Description of research objectives.
- Prevalence of *Leptospira* as the main objective.
- Sampling methodology.
- Period of study.
- Diagnostic method.
- Use of immunological, serological or molecular techniques.
- Categorization of subjects (age, sex), HIV infected and HIV uninfected.
- Representability of target sample in the general population.
- Random selection of samples.
- Sample size.

The main outcome was to: a) determine the prevalence of *Leptospira* in HIV uninfected people and in PLHIV from SADC countries; b) describe the sociodemographic, clinical and epidemiological characteristics of *Leptospira* infection retrieved from the studies analyzed; c) and describe the diversity of genotypes and serovars of circulating *Leptospira* spp. in the studies. The secondary outcome was to assess the existing gaps in knowledge in this area and to better describe research priorities.

### Data analysis

Pooled prevalence of *Leptospira* spp. in humans within SADC countries and 95% confidence intervals (CI) were determined and expressed in forest plots in the STATA program. Variation between studies were expressed by Inverse variance index (I^2^) with values of 25%, 50% and 75% classified as low, moderate and high degree of heterogeneity, respectively.

## Results

### Prevalence and diversity of Leptospira

**Fig 1:**
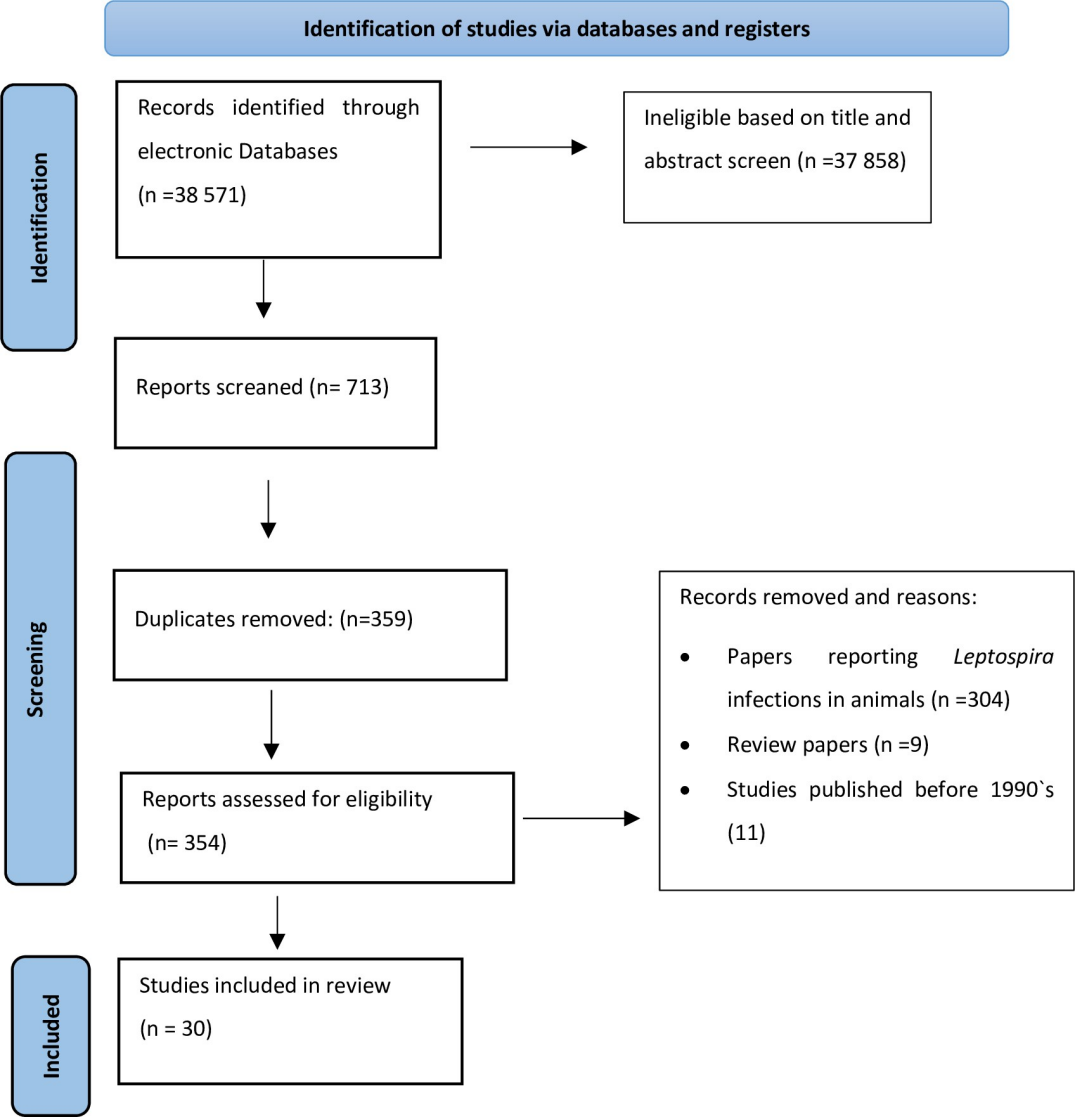
Selection criteria of literature used for the present study.

**Fig 2:**
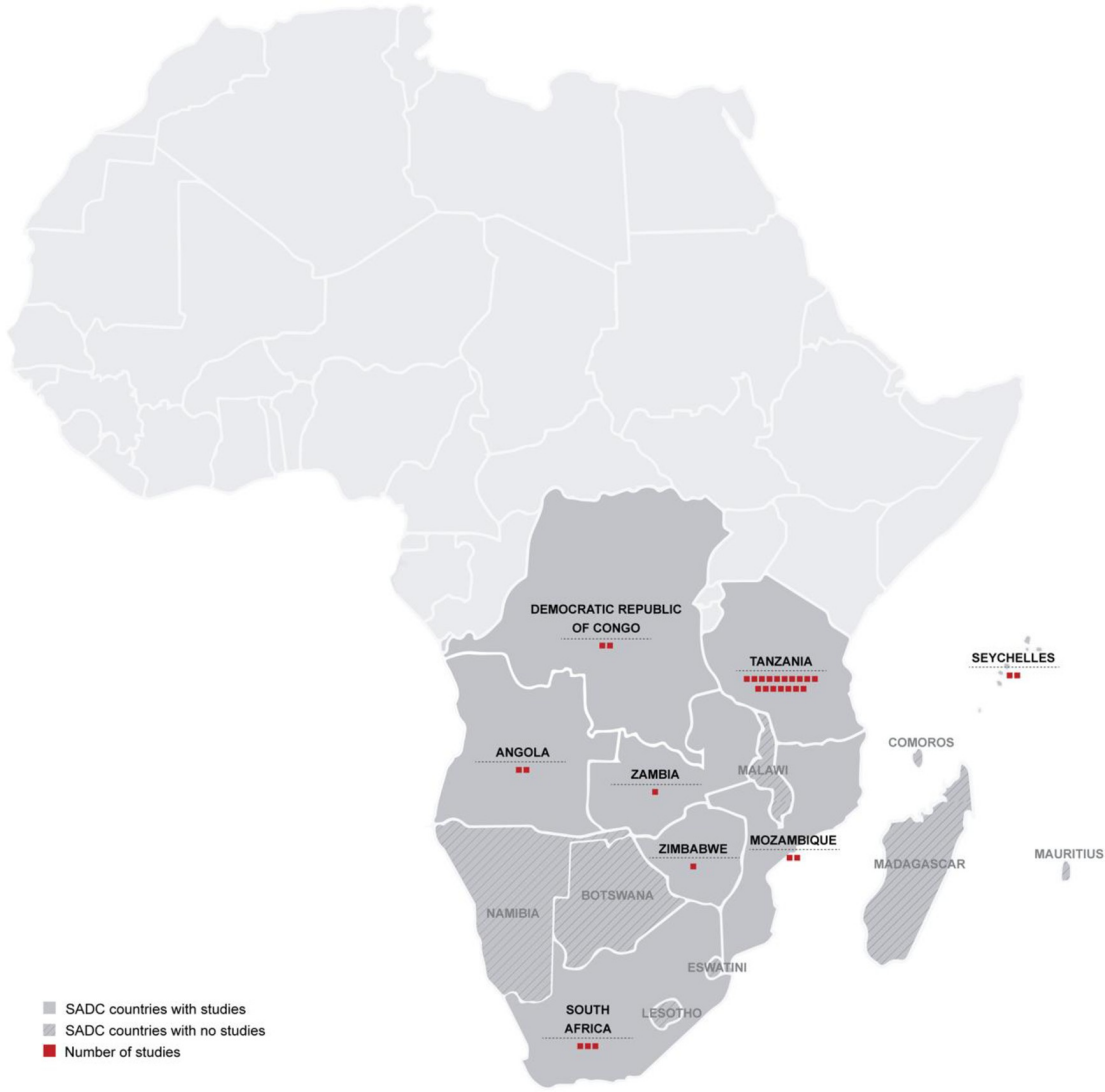
Distribution of *Leptospira* studies from the SADC countries. Created by Patricia Noormahomed based on LandsatLook (https://landsatlook.usgs.gov/explore).

Table 1 summarizes the number of studies identified by country and for each study, the prevalence of leptospirosis, diagnostic methods, period of the study, quality and index scores. In total we identified thirty (30) studies (Fig 1) from eight (8) out of sixteen (16) SADC countries, within our parameters. Amongst them, 18 were community based and 12 were hospital-based. Studies reporting prevalence values of *Leptospira* in humans were from Angola (2), Democratic Republic of Congo (2), Mozambique (2), Seychelles (3), South Africa (3), Tanzania (16), Zambia (1), and Zimbabwe (1). No studies were obtained from Botswana, Comoros, Lesotho, Madagascar, Malawi, Mauritius, Namibia, and Eswatini (Fig 2). The most used diagnostic method was the MAT (23) used alone or in combination with either a serological (immunological-ELISA) or molecular (PCR) tool. Seven studies used ELISA alone and six studies used only PCR.

**Table 1:** Prevalence of Human leptospirosis in the SADC countries.

Countries	Np	n	P (%)	Diagnostic test	Study period	Quality Score	Index score	Reference
**Angola**	141	14	9.9	MAT, MACROLepto and PCR	2011	8	0.8	[40]
	130	62	47.7	MAT	2015	8	0.8	[41]
**Mozambique**	373	43	11.5	ELISA, MAT	2012–2015	8	0.8	[42]
	160	14	8.8	MAT	1993	5	0.5	[43]
**DRC**	54	29	53.7	MAT	2004–2005	6	0.6	[44]
	1300	88	7	ELISA	2017–2018	8	0.8	[45]
**Seychelles**	223	51	22.9	PCR, IgM ELISA and MAT	2014–2015	9	0.9	[36]
	125	75	60.0	MAT and PCR	1995–1996	8	0.8	[46]
	80	58	73.0	MAT	1988–1990			[47]
**South Africa**	219	41	19	Agglutination test	2004–2005	7	0.7	[48]
	217	43	19.8	IgM ELISA	2003–2006	4	0.4	[49]
	138		21.9	ELISA				[50]
**Tanzania**	1293	252	19.5	MAT	2012–2014	5	0.5	[17]
	831	70	8.4	MAT	2007–2008	8	0.8	[26]
	200	26	13	ELISA	2013	8	0.8	[31]
	870	40	4.5	MAT	2007–2008	8	0.8	[32]
	453	40	8.8	MAT	2007–2008	7	0.7	[35]
	1017	12	1.2	MAT	2012–2014	6	0.6	[37]
	588	42	7.14	MAT	2011	8	0.8	[38]
	267	80	29.96	MAT	2012–2013	9	0.9	[51]
	267	80	29.96	MAT, PCR	2013–2014	8	0.8	[52]
	375	1	0.3	MAT	1994–1996	6	0.6	[53]
	455	72	15.8	MAT	2019	4	0.4	[54]
	250	25	10	MAT	2017	5	0.5	[55]
	199	30	15.1	MAT	2005	7	0.7	[56]
	128	3	2.3	PCR	2016	7	0.7	[57]
	50	15	30	MAT	2016	8	0.8	[58]
	111	4	3.6	MAT, PCR	2007-20082012-2014	7	0.7	[59]
**Zambia**	282	65	23.0	DFM and ELISA IgG and IgM	2014	8	0.8	[60]
**Zimbabwe**	182	152	83.5	MAT		7	0.7	[61]

Np: number of study participants, n: number of participants who tested positive, P: prevalence of leptospirosis, DRC: Democratic Republic of Congo.

**Fig 3:**
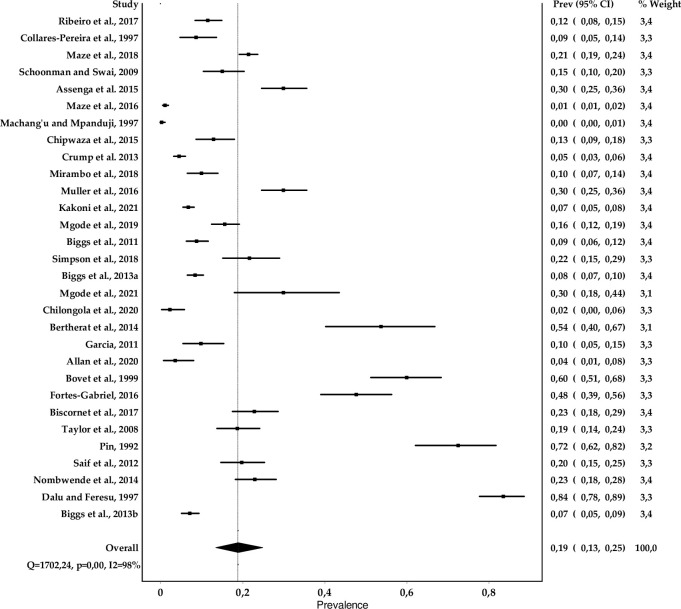
Forest plot of prevalence estimates of *Leptospira* spp. in humans with random effects analysis in SADC countries.

**Fig 4:**
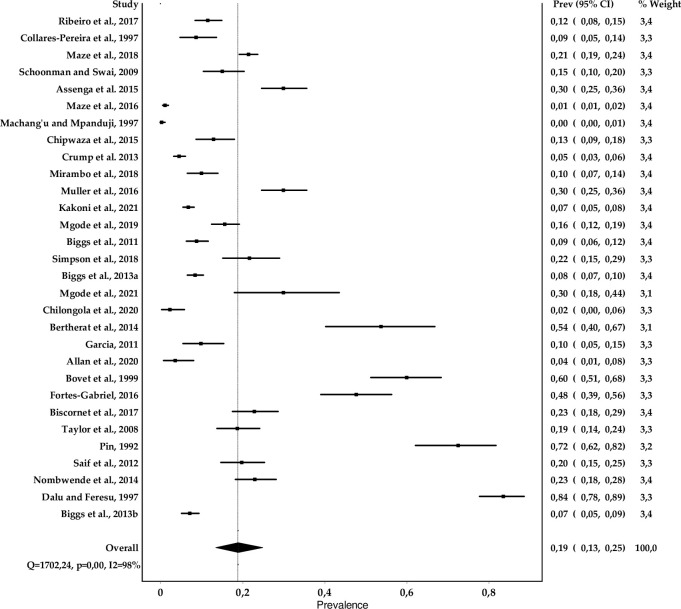
Forest plot of prevalence estimates of human leptospirosis from Tanzania.

Fig 3. shows the forest plot for the prevalence of *Leptospira* spp. from the reviewed studies. We found an overall prevalence of 19% (CI: 13–25%) with variations from 0 to 83.5% between countries, heterogeneity level of 96% and score index ranging from 2 to 9. Tanzania alone contributed to 16 (53.3%) of the studies in the region. When analyzing the pooled prevalence of studies conducted in Tanzania we found a pooled prevalence of 10% (6–16%) as shown on Fig 4.

With regard to leptospirosis in PLHIV, our review found only four studies from Tanzania (3) and Zambia (1) and the prevalence of the co-infection varied between 4.4% and 33% [26,32,35,60].

Table 2 summarizes demographic, clinical and risk factors for human leptospirosis. As noted in the table, the studies were conducted in both male and female patients, most of them presenting with febrile and other non-specific signs and symptoms. In some of the studies, other pathogens or conditions associated with fever were screened, such as *Plasmodium* spp. [17,31,32,35,57], *Brucella*, *Rickettsia* [32,35,55,62] and typhoid fever [31,35] for differential diagnosis.

**Table 2:** Descriptive summary of socio demographic, clinical epidemiological characteristics of *Leptospira* infection retrieved from the studies included in the review.

Countries	Study objectives	Study type	Population characteristics	Outcomes/Conclusions	Reference
**Angola**	To investigate the occurrence of leptospirosis in Lobito (Benguela Province) and identify circulating serovars of *Leptospira* interrogans sensu lato (l.s.), using serological and molecular techniques.	Cross-sectional study	141 Febrile patients (64 males and 77 females) with headache, myalgia and nausea	There were confirmed *Leptospira* infections in the region which may contribute to the inclusion of the disease in the list of febrile diseases in Angola and other tropical countries.	[40]
**Mozambique**	To investigate the occurrence of leptospirosis in febrile patients.	Cross-sectional study	Febrile patients, median age of 12 and 33 years, 171 male and 202 female.	Leptospirosis was prevalent among Mozambicans, and most cases were misdiagnosed as malaria.	[42]
	To evaluate the importance of *Leptospira* and *Borrelia* as causes of human diseases in Mozambique	Cross-sectional study	160 febrile adult patients aged between 18 and 50 years old, 63 were males and 97 females. Myalgia, headaches, chills, anoxia, cough (3 patients), arthralgia (2), chest pain (1)	Females were more infected than males. Leptospirosis was underdiagnosed due to its non-specific presentation and to lack of laboratory facilities for specific diagnosis.	[43]
**DRC**	To determine the seropositivity of anti-*Leptospira* antibodies among suspected yellow fever cases and map the geographical distribution of possible leptospirosis in the DRC.	Retrospective	1300 participants suspected to have yellow fever with acute fever, jaundice and not responding to antimalarial drugs. 58.2% were Maleand 41.8% female. *Leptospira* was more likely to affect male (79.6%) than female (20.4%). Most cases were from urban (59.1%) than from rural area (40.9%) and cases where frequent in rainy season (82.9%). People with anti-*Leptospira* antibodies ranged in age from 4 months to 86 years old, with a median age of 16 years. Thirty leptospiral IgM positive cases (34%) were found among the 20–29 years age group, followed by the 0–9 years age groups with 20 (23%) of positive cases	Leptospirosis is likely an overlooked cause of unexplained cases of -fever with jaundice in the DRC and highlights the need to consider leptospirosis in the differential diagnosis of fever with jaundice, particularly in young adult males	[45]
	To investigate cases of Plugue and *Leptospira* during outbreak of lethal pneumonia	Prospective survey	10 female and 19 male with pneumonia	While plague remains the main culpable agent for the outbreak of severe pneumonia in the miners’ camp, a leptospirosis outbreak co-existed.	[44]
**Seychelles**	To determine the current burden of leptospirosis in Seychelles, establish epidemiological links between animal reservoirs and human disease, and to identify drivers of transmission.	Prospective population-based survey	Patients aged above 13 years with fever of 38°C for more than three days with or without any of the following signs and symptoms: headaches, myalgia and hemorrhagic manifestations. There were 23 females and 198 males with a mean age of 33 years old (range 13 years– 60 years).	Human leptospirosis still represents a heavy disease burden and there is limited efficacy of preventive measures so far implemented in Seychelles. This could result from ineffective control measures of excreting animal populations, possibly due to a misidentification of the main contaminating reservoir(s).	[36]
	To investigate the frequency and associated factors of leptospirosis	Case-control	Male and female patients who had fever of unknown origin with any of the following symptoms: myalgia, tender liver, meningism, bleeding tendency, jaundice, acute renal failure, radiological lungs infiltrates, renal failure, jaundice and pulmonary haemorrhage.	There was a high incidence of leptospirosis in Seychelles suggesting that leptospires were likely to be ubiquitous	[46]
**Tanzania**	To investigate risk factors for acute leptospirosis and *Leptospira* seropositivity among patients with fever attending referral hospitals in northern Tanzania.	Cross-sectional study	Patients had an axillary temperature of >37.5°C or a tympanic, oral, or rectal temperature of ≥38.0°C.	Exposure to cattle and rice farming were risk factors for acute leptospirosis.	[17]
	Identify the prevalence of leptospirosis, brucellosis, typhoid fever and urinary tract infections and their rate of co-infections with malaria.	Cross-sectional study	Febrile children aged from 2–13 years, axillary temperature ≥37.5°C or rectal temperature ≥38.0°C at the time of recruitment.	Leptospirosis, brucellosis, typhoid fever and urinary tract infections should be considered by clinicians in the differential diagnoses of febrile diseases. However, access to diagnostic tests for discrimination of febrile illnesses is needed.	[31]
	Describe comprehensively the causes of febrile illness in northern Tanzania among patients sufficiently ill to require hospitalization	Cohort—Prospective	Infants and children aged from ≥2 months to <13 years, with a history of fever in the past 48 h or an axillary temperature ≥37.5°C or a rectal temperature of ≥38.0°C; adolescents and adults aged ≥13 years and with oral temperatures of ≥38.0°C	Malaria was uncommon and over-diagnosed, whereas invasive infections were underappreciated. Bacterial zoonoses and arbovirus infections were highly prevalent yet overlooked.	[32]
	To assess the importance of leptospirosis as a causative of febrile illness in Tanzania	Cohort—Prospective	467 febrile pedriatic patients (≥2 months to <13 Years old) and 403 adult patients (≥13 years old). Median age among participants with confirmed or probable leptospirosis was 23.3 years: 39.8 years among adults and adolescents and 3.1 years among infants and children Symptoms: rigors, headache, cough, jaundice, thrombocytopenia and Lymphopenia	Five (7.1%) of 70 participants with evidence of leptospirosis died (2 had HIV infection, 2 had documented diabetes and 1 had cirrhosis of the liver). Of died patients, 2 got infections by *Escherichia coli* and 1 with *Streptococcus pneumoniae*. The study suggests that livestock were important reservoirs for *Leptospira* being a major yet underdiagnosed cause of febrile illness in northern Tanzania	[35]
	To estimate the incidence of acute leptospirosis in Kilimanjaro Region, northern Tanzania for the time period 2012–2014	Cross-sectional study	Of the 1,115 participants, 409 (37.7%) were <5 years, 111 (10.0%) 5–14 years, and 595 (53.4%) were 15 years old. A total of 593 (46.9%) participants were male and 758 (74.6%) participants had fever for at least 3 days. Adult and pediatric patients with history of fever within the previous 72hours or an axillary temperature of >37.5°C or a tympanic, oral or rectal temperature of 38.0°C at admission.	The study indicated a dynamic epidemiology of leptospirosis in Kilimanjaro highlighting the value of multi-year surveillance to understand leptospirosis epidemiology	[37]
	To evaluate if HIV patients were at increased risk of infection with leptospirosis and severity of symptoms.	Cohort—Prospective	Febrile pedriatic patients (≥2 months to <13 Years old) with an axillary temperature of ≥37.5°C or a rectal temperature of ≥38.0°C. and adult patients (≥13 years old) with an oral temperature of ≥38.0°C The median age of those with HIV and leptospirosis was 31.4 years. 55.6% of HIV patients co-infected by *Leptospira* were male Median (range) CD4 count of those with leptospirosis and HIV infection was 335 cells/lL, and the median (range) CD4 percent was 20.	Among HIV-infected patients, those with leptospirosis were not more immunosuppressed relative to those with other etiologies of febrile illness.	[38]
	To determine the seroprevalence of *Leptospira* spp in humans, domestic ruminants and wildlife.	Cross-sectional study	Households with domestic animals (children below the age of 2 years were excluded)	There were common serogroups circulating among humans, domestic ruminants and wildlife	[51]
	To explore the genetic characteristic of *Leptospira* species which are prevalent among agro-pastoralists living in Katavi–Rukwa Ecosystem, Tanzania.	cross-sectional study	Agropastoral community: children (age 2 to < 13 years), and adults (age ≥13), the majority were adults (59.5%) and others were children (40.4%). Adults were more likely to be infected (63.7%) comparing to children (36.2%). There were more male patients infected (56.2%) comparing to female (43.7%). 9	Molecular techniques have confirmed the presence of pathogenic *Leptospira* species circulating among agro-pastoralists. The presence of *Leptospira* species poses a public health threat to the communities.	[52]
	To determine the seroprevalence of *Leptospira* in rodents, cattle, dogs and humans in selected areas os Tanzania	Cross-sectional study	Assymptomatic 159 adult male cane cutters. No data available for other 216 patients.	*Leptospira* was a potentil public health hazard in certain areas of Tanzania	[53]
	To determibe the prevalence of antibodies against *Leptospira* in sugarcane plantation workers, fishing community, rodents and shrews in the Kagera region, northwestern Tanzania.	Cross-sectional study	455 adult sugarcane workers and fishing community of which 401 (132 females and 269 males) were from sugarcane plantation and 54 (16 females and 38 males) were fishing community. *Leptospira* seropositiviy was as follows: 18–37 years old (14.5%), 38–57 (16.7%), ≥58 (54.5%).	The study group was affected by *Leptospira* and public awareness targeting risk occupational groups is much needed for mitigation of leptospirosis in the study areas and other vulnerable populations in Tanzania and elsewhere.	[54]
	To investigated seropositivity of *Brucella* spp. and *Leptospira* spp., and associated factors among abattoir workers and meat vendors in the city of Mwanza, Tanzania	Cross-sectional study	Abattoir workers and meat vendors. Median age of participants was 31 Only 0.4% were females, and 84.8% of the participants were from urban areas.	Seropositivity of *B*. *abortus* antibodies among abattoir workers and meat vendors was high and a significant proportion of abattoir workers and meat vendors w seropositive for *Leptospira kirschneri* serovar Sokoine	[55]
	To estimate the seroprevalences of human infection with various *Leptospira* serovars among apparently healthy inhabitants of Tanga city, Tanzania	Cross-sectional study	The mean age of the 199 subjects investigated (132 males and 67 females) was 36.6 years, with 162 (81%) of the subjects aged 20–50 years.	There was evidence of widespread environmental contamination with pathogenic leptospires, and of some occupation-specific exposure	[56]
	To understand circulating *Leptospira* serovars and potential major reservoirs	Cross-sectional study	-	Livestock, especially goats and sheep, could be the major source of leptospirosis transmission to humans.	[58]
	To determine the prevalence and distribution of *Plasmodium*, *Leptospira* and *Rickettsia* infections in northern Tanzania.	Cross-sectional study	64.8% were above 15 years old while 16.4% among them were children below five years. Nearly two-thirds or 66.4% were females with only 8.1% of all participants found to have fever at the time of survey. *Leptospira* infection was observed to occur in the age group of >15 years, with higher proportion (66.7%) in male than females	*Plasmodium* was identified as the main cause of fever. While *Plasmodium* and *Leptospira* contribute to fevers, Rickettsia infection was an insignificant cause of fever in Northern Tanzania.	[57]
	To characterize the genetic diversity of human *Leptospira* infection in northern Tanzania and to infer possible sources of human infection in Tanzania	Cross-sectional study	Adult and pediatric hospitalized with fever	There was a close relationship between *Leptospira* genotypes found in people and livestock.	[59]
**Zambia**	To assess the distribution of Leptospirosis in PLHIV and ascertain the necessary health care for the disease in Zambia	Cross-sectional study	150 PLHIV on ART and 132 non-HIV (controls) aged between 25–34 years old. 23% were females and 10% males	HIV patients despite being on ART had higher chances of contracting leptospirosis	[60]
**Zimbabwe**	To determine the role domestic rodents play in transmitting leptospirosis on two City of Harare farms	Cross-sectional study	Farm workers and members of their families.	Leptospirosis was a common occupational disease of workers on the two farms which is transmitted to them by rodents.	[61]

Pediatric (≥2 years to 13 years old) and adult patients who were up to 60 years old were included. Median age of *Leptospira* patients was of 3.1 years among infants and children and 39.8 years among adults. *Leptospira* more likely infected male (56.2–79.6%) than female patients (20.4%-43.7%) and adults were more likely to be infected (63.7%) compared to children (36.2%).

PLHIV included in reviewed studies aged between 25–34 years. In this group, male patiens were also most likely to be infected by *Leptospira*. The median age of those PLHIV was 31.4 years and the median CD4 cell count was 335 cells/μL. Patients on ART had higher chances of contracting leptospirosis than HIV uninfected patients. PLHIV co-infected with *Leptospira* were not more immunosuppressed comparing to those with other febrile etiologies and mortalities in PLHIV were atributed to other ethiologies [38].

Overall, the studies included a variety of households, associated or not with domestic animals, sugarcane plantations, fishing communities, abattoir workers, meat vendors, prisoners and farm workers.

The studies showed, that besides malaria as expected, leptospirosis is also a potential cause of febrile illness, however, it is under-reported in comparison with malaria [32,42,57]. In addition, *Leptospira* infections were reported to be associated with contact with rodents and livestock (cattle, goats, and sheep). We also noted a relationship between *Leptospira* genotypes circulating in humans and livestock, with reports of pathogenic species circulating among farm workers [17,51,58,59].

### *Leptospira* species and serogroups

Table 3 summarizes the species and serogroups of *Leptospira* identified as well as the method used. There was a total of 23 studies: Angola (2), Mozambique (2), Seychelles (2), Tanzania (14) and Zimbabwe (3) in which identification of either *Leptospira* species (6), serogroups (20) or both (3) were done. Only three species of *Leptospira* were identified as follows: *Leptospira interrogans* (4), *L*. *kirschneri* (3) *and Leptospira borgpetersenii* (1). Concerning the identification of serogroups, 23 were found, the most frequently being *Icterohaemorrhagiae* (13), *Australis* and *Grippotyphosa* (10) followed by *Sejroe* (8).

**Table 3:** Species and serogroups of *Leptospira* Identified in study participants from the SADC countries.

Country	Species	Serogroups	Diagnostic test	Authors
**Angola**	*L*. *interrogans*	Icterohaemorrhagiae (Copenhageni), Australis (Bratislava) and Sejroe serogroups	PCR, MAT	[40]
		Javanica, Australis, Panama, Louisiana, Ballum	MAT	[41]
**Mozambique**		Australis, Icterohaemorrhagiae	MAT	[42]
		Ballum, Javanica, Panama, Mini, Louisiana, Icterohaemorrhagiae	MAT	[43]
**Seychelles**	*L*. *interrogans*	Icterohaemorrhagiae, Autumnalis, Hurstbridge, Australis, Djasima, Sejroe.	PCR, MAT	[36]
		Icterohaemorrhagiae, Autumnalis	MAT	[47]
**Tanzania**		Icterohaemorrhagiae, Sejroe, Grippotyphosa, Hebdomadis, Australis, Celledoni, Pyrogens, Mini, Betaviae, Autumalis, Djasiman, Tarrassovi	MAT	[17]
		Sokoine, Kenya, Gripotyphosa, Hebdomadis, Lora	MAT	[31]
		Icterohaemorrhagiae, Mini and Australis	MAT	[35]
		Australis, Sejroe, Grippotyphosa, Icterohaemorrhagiae, Pyrogenes, Djasiman, Tarassovi	MAT	[37]
		Canicola, Icterohaemorrhagiae, Bataviae, Hebdomadis, Ballum and Sejroe	MAT	[44]
		Icterohaemorrhagiae, Grippotyphosa, Sejroe	MAT	[51]
	*L*. *interrogans and L*. *kirschneri*	Icterohaemorrhagiae, Sejroe, Grippotyphosa, Hebdomadis, Australis, Ballum	PCR, MAT	[52]
		Grippotyphosa	MAT	[53]
		Lora, Sokoine, Pomona, Hebdomadis, Kenya	MAT	[54]
		Sokoine and Grippotyphosa, Lora	MAT	[55]
		Icterohaemorrhagiae, Bataviae, Hardjo, Tarrassovi, Ballum, Pomona	MAT	[56]
		Icterohaemorrhagiae, Grippotyphosa, Australis	MAT	[58]
	*L*. *interrogans*, *L*.*borgpetersenii*, *L*. *kirschneri*		PCR	[59]
		Australis, Sejroe, Icterohaemorrhagiae, Djasiman	MAT	[62]
**Zimbabwe**		Icterohaemorrhagiae, Pyrogenes and Grippotyphosa	MAT	[61]
	*L*. *kirschneri*	Pomona and Grippotyphosa	PCR, MAT	[63]
		Tarassovi	PCR, MAT	[64]

## Discussion

To the best of our knowledge, this is the first systematic study aiming at reviewing SADC information regarding the epidemiology of *Leptospira* infection in both HIV uninfected people and in PLHIV, taking into account sociodemographic and clinical characteristic of participants, as well as defining the diversity of circulating *Leptospira*. We also sought to uncover gaps in knowledge and recommend research priorities. From 38571 manuscripts retrieved, we were only able to include 30 studies, confirming scarcity of data on the subject. We found that most studies were conducted in Tanzania (16/30) where a pooled prevalence of 19% (CI: 13–25%) of *Leptospira* infections was found. Overlapping prevalence rates were found in studies of other African regions as follows: in West Africa (Nigeria and Senegal) a prevalence of 7.7% - 20.4% [65,66], in Central Africa (Gabon, Democratic Republic of Congo) a prevalence of 7% - 15.7% [45,67], and in East Africa (Kenya, Ethiopia) a prevalence of 26–47.5% [68,69]. Similar heterogeneity with a variation from 10% to 88% was observed in other regions of the world such as the Pacific Islands and Jamaica [1,70].

Tanzania, with a larger number of studies had a median prevalence of leptospirosis of 10%, well below the pooled prevalence for the SADC region, though more than half of incidence and outbreaks were found in this country. It is difficult to explain why most of the studies were done in Tanzania. It is well documented that in low-income countries, the research agenda is often guided by funding opportunities, apart from the individual interest of researchers and institutions involved. This could be one of the reasons for such discrepancies within different SADC countries [71]. We also found that among the four (4) available studies about HIV- *Leptospira* co-infection, three (3) were from Tanzania and one was (1) from Zambia. The reported prevalence in PLHIV varied from 4.4% in Tanzania, to as high as 33% in Zambia. In the study from Zambia, the authors concluded that PLHIV had higher chances of contracting *Leptospira* infection compared to HIV uninfected participants [60] while a study done in Tanzania concluded otherwise. Further, in the studies from Tanzania the authors concluded that *Leptospira* infections were not associated with increased HIV immunosuppression [35]. These conflicting results may be attributed to the research design which included sociodemographic and clinical characteristics of the study participants, nuances of case definition and, above all, the onset date of symptoms at the time of screening for diagnosis, (which would affect the final comparison of results). Further, MAT may have used different panels of antigens, resulting in differences in sensitivity and specificity and that may also be affected by the presence of other bacteria species [1,35,38,42,52]. In adition, PLHIV on antiretroviral therapy (ART) are more likely to go to health units for care, and this may explain why in the study from Tanzania, in patients in ART, were less likely to be infected by *Leptospira* [35].

PCR is the more sensitive and specific tool to detect the infection compared to the cornerstone MAT, but its use for routine diagnosis is limited due to its complexity and expense. Direct observation of *Leptospira* is challenging due to the size of the bacteria to be detected by ordinary microscope, but it can be effective by darkfield or phase-contrast microscopy, immuno-peroxidase staining and direct immunofluorescence. However, low sensitivity (40.2%) and specificity (61.5%) can induce both false negatives and false positives [72]. Thought the MAT test is less sensitive in early stage of the infection, its sensitivity increases from 41% to 96% between the first and the fourth week of illness. The test is often available in reference laboratories, and thus allows the detection of specific antibodies serogroups and serovars [73]. The IgM Enzyme-Linked Immunosorbent Assay (ELISA) can be used as an alternative to the MAT test, with sensitivity varying from 52% to 96.6% and a specificity of 93.3% [72,74].

We found that *Leptospira* was more likely to affect male than female patients and that adults were more likely to be infected than children. Similar results were reported in another review of leptospirosis in which adult males were also found to be more likely to get infected by *Leptospira* than females [2]. We may associate these findings with certain occupational activities that are mainly practiced by men [1,2,8].

After reviewing the studies for this work, we confirmed that a high number of negative results on malaria diagnosis by microscopy or rapid diagnostic testing (20% to 80%), with a confirmed *Leptospira* infection were treated with anti-malarial drugs, or sometimes with a combination with antibiotics [28,31,35,42,63,75]. This approach is valid in many African countries and, elsewhere as in Jamaica [70,76]. Though malaria cases are decreasing globally [24] including in some SADC countries, we can expect that a significant fraction of febrile patients are wrongly diagnosed with malaria at the expense of leptospirosis [17,77,78].

This review also exhibited the scarcity of detailed information in the region about circulating species, serogroups and serovars, and their relationship with disease severity and outcomes, host reservoir and sources of infection. Little is known on the risk factors associated with transmission to humans of this zoonotic and neglected disease. Despite the limited studies, the review revealed that contact with rodents, cattle, and pigs were the most frequently associated risk factor for human infection, confirming its zoonotic nature [79,80]. Furthermore, the Grippotyphosa serogroup was almost always isolated from cattle while the Icterohaemorrhagiae group was most frequently isolated from rodents, cattle and pigs, reflecting the wide range of reservoirs and sources of infection for humans. Less predominant subgroups such as Pomona and Sejroe were also isolated from cattle [81].

In general, paucity of studies, lack of diagnostic resources, lack of an active surveillance system and awareness of health professionals and authorities about the disease is also valid for other African countries outside SADC [82]. In addition, excessive attention paid to diseases such as HIV, malaria and tuberculosis has lead to underestimation and perpetuation of the neglected status of this and other neglected tropical diseases which are also predominant in this region [5,13,83,84]. In view of this, a One Health approach studies that includes the study of *Leptospira* species, serogroups and serovars, should be carried on, in combination with studies on the epidemiology of the bacteria in humans and animals identifying host range involved in transmission cycles and factors associated with infections [2]. This is especially relevant in the context of climatic changes where we expect adverse events like floods, heavy rainy falls, which will increase sources of contamination to humans [1]. The expected population growth and urbanization associated with poor sanitation, environment contamination will also contribute to further increase in the incidence and prevalence of leptospirosis.

Our study has some limitations that should be highlighted. First, we could not find data on the morbidity and mortality atributed to *Leptospira* infection in the region. There was also an absence of studies in about half of the SADC countries, so it is not possible to draw firm conclusions on the real burden of the disease.

Secondly, in many low and middle income countries, empiric administration of antibiotics in the absence of clear diagnosis is reported [28,31,35,42,76] and this may have contributed to the underestimation of leptospirosis. Prevalence values presented in our review should be interpreted with caution.

In summary, this review confirms that SADC countries, as elsewhere, are still lacking data on the epidemiology and clinical features of leptopirosis, alone or in relation to HIV. As malaria, tuberculosis and HIV are the leading causes of morbidity and mortality in the region, since much attention and funding has been used for diagnosis and management of these infections, we believe that the impact of leptospirosis is under estimated. Therefore, we recommend more investments to address the burden of this and other neglected tropical diseases, as well as to strengthen prevention, control and treatment measures. Furthermore, as HIV infection often worsens or predisposes to acquisition of other diseases, systematic studies on interactions of these diseases and HIV are required.

Studies of associations and comparisons among diseases also demand standardization of techniques. This is also critical in relation to the development of accurate classifications of pathogenecity and morbidity caused by *Leptospiras*.

It is our hope that the gaps in knowledge observed in this review can be a good starting point for researchers in the region and can contribute at national and inter-SADC levels to motivating better studies of leptospirosis in our region.
